# Case report of a successful multidisciplinary approach to a giant scrotal malformation

**DOI:** 10.1016/j.ijscr.2020.03.019

**Published:** 2020-03-28

**Authors:** A. Ascoli Marchetti, G. Citoni, R. Gandini, A. Ippoliti

**Affiliations:** a Vascular Surgery Unit Biomedicine Department, University of Rome Tor Vergata, Italy; b Radiology Unit, University of Rome Tor Vergata, Italy

**Keywords:** Selective angiography, Embolization, Giant arteriovenous malformation, Previous surgery, Multidisciplinary

## Abstract

**Introduction:**

Congenital pelvic malformations are rare and represent a difficult therapeutic challenge. Scrotal arteriovenous malformations are quite unusual, with only a few such cases reported in the literature. Only one case of scrotal malformation? due to an arteriovenous fistula resulting in azoospermia has been described.

**Presentation of case:**

The two-phase strategy adopted in that case permitted complete treatment of a large-sized malformation that was served by 4 main blood confluences.

**Discussion:**

Angio-CT performed using reconstructions with MIP and SSD algorithms provided more detailed data about the extension of the afferents and efferents of the arteriovenous malformation, thus enabling us to plan the endovascular treatment of the lesion.

**Conclusion:**

A multidisciplinary approach is mandatory to achieve good results in these cases.

## Highlights

• Giant arterio-venous malformations in the pelvic region are rare.
• Selective angiography is mandatory for diagnosis and treatment to define the extent and limits of the malformation.
• Skin necrosis can by managed jointly by vascular and plastic surgeons.
• Azoospermia is frequent in the postoperative period.

## 1 Introduction

This paper is being reported in line with the SCARE criteria [1]. Congenital pelvic malformations are rare and represent a difficult therapeutic challenge. Scrotal arteriovenous malformations are quite unusual, and only a few such cases have been described in the literature. Due to their complex pathology and a lack of available data, the diagnosis and treatment of this type of cases require a multidisciplinary approach to treat the lesion and preserve both the fertility and the sexual functionality of the patient.

## 2 Clinical case

**Fig. 1:**
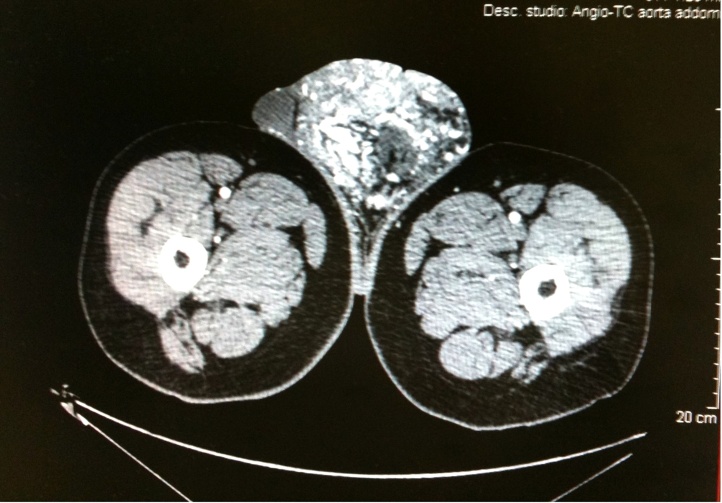
Angio-CT MIP of the lower abdomen and the cranial portion of inferior limbs: See the presence of a raw agglomerate of enlarged blood vessels located in the left-side of the scrotum and contralateral dislocation of the right testicle.

**Fig. 2:**
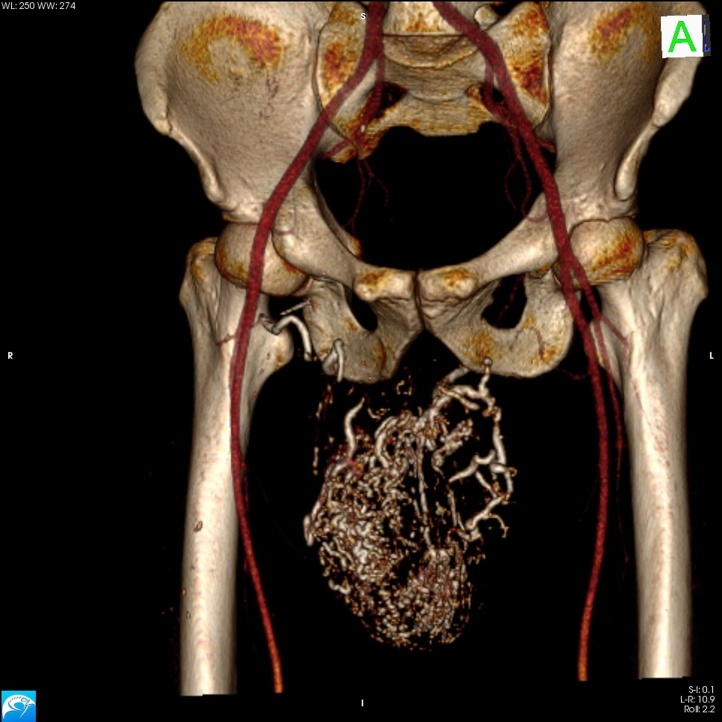
Angio-CT SSD reconstruction swowing normal vasculararisation of the iliofemoral area and the selective embolisation with Glue deposition in the A-V malformation and in the arterial ramifications coming from the right femoral artery.

**Fig. 3:**
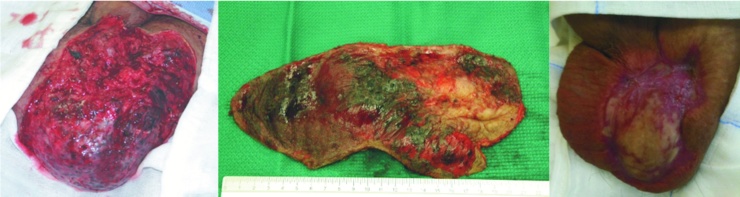
A. Right hemiscrotum aspect after necrotic tissue asportation. B. Necrotic skin tissue removed. C. Six monts appearance after the procedure. Note the volume reduction of the scrotum.

At the Vascular Surgery Department of our Institution, we examined a male patient (42 years old) who presented with a scrotal tumefaction. This tumefaction was approximately 15 × 17 cm in size and had been progressively developing after surgery for the removal of a left-side testicular angioma. Secondary sterility had occurred, as suggested by a spermiogram that revealed azoospermia (<20,000 spermatozoa/mL). Ultrasonographic test results performed during hospitalization revealed a diagnosis of scrotal arteriovenous malformation and a small fluid slope in the left tunica vaginalis; the testicles appeared to be regular in shape and had an echographic pattern. The patient underwent an angio-computed tomography (CT) of spiral multislides of the abdominal aorta and the lower limbs. The exam results indicated the presence of a raw agglomerate of enlarged blood vessels located in the left-1 side scrotal portion that was causing a contralateral dislocation of the right testicle (Fig. 1). The mass was supplied by four arterial confluences: two from the superficial femoral arteries and two from the hypogastric arteries through the bilateral penile arteries. In addition, the scrotal venous system showed varicosity and congestion of the bilateral common femoral veins, most likely due to an arteriovenous shunt. It was decided that the malformation should be treated on the basis of a protocol involving endovascular arterial embolization with Glubran 2. The patient, after signing his informed consent, underwent a selective catheterization of the arterial ramifications via a left-side percutaneous transfemoral approach originating from the left femoral and left hypogastric arteries. A postprocedural angiographic check indicated devascularization of the lesion and a slight reduction in the size of the scrotal tumefaction. Two days after the procedure, a second embolization was performed via a left transfemoral approach to obtain a further reduction in the vascularization of the tumefaction, combined with the selective catheterization of the arterial ramifications coming from the right femoral and right hypogastric arteries (Fig. 2). After 72 h from the embolization a skin necrosis on the hemiscrotum due to ischemia of the area previously supplied by the anomalous blood vessel was present. To avoid the development of a serious form of infected necrosis, such as Fournier’s gangrene, the patient underwent repeat surgery and debridement of the wide necrotic skin tissue (Fig. 3A B). During the following days, the patient received daily medications at the lesion site, coupled with endovenous antibiotic therapy. The agglomerate progressively decreased in size, and the necrotic area increased its demarcation. The patient was discharged from the hospital with antibiotic therapy 20 days after the first embolization procedure. In addition, he was prescribed daily medications, and he underwent subsequent plastic surgery for scrotal reconstruction. The spermiogram result showed azoospermia in both testicles.

## 3 Discussion

Testicular arteriovenous malformations are very rare lesions that represent less than 1% of vascular neoformations; they can be congenital, traumatic or iatrogenous [[2], [3], [4]]. Because of the rarity of this pathology, it is not always easy to obtain an accurate diagnosis or proper treatment. Typically, the treatment includes surgical knots or the embolization and successive removal of the agglomerate [5]. In the case described here, the patient’s condition was complicated by a previous operation that had been performed to remove a scrotal angioma. After that surgery, a progressive and significant increase in the size of the lesion was observed until it reached a large size and impacted the patient’s fertility [6]. Thus, it is fundamental to obtain a correct and precise evaluation of the nature and type of vascularization of this type of lesion in order to intervene appropriately, thus preventing recurrence and preserving, as much as possible, the healthy surrounding tissue so that deleterious effects in the sexual and reproductive spheres can be avoided. Angio-CT performed using reconstructions with MIP and SSD algorithms provided more detailed data about the extension of the afferents and efferents of the arteriovenous malformation, thus enabling us to plan the endovascular treatment of the lesion. Due to the large size and position of the agglomerate, it was decided that the embolization would be completed in two phases so as to minimize the risks and ischemic complications entailed in the operation, such as possible additional fertility damage, oerigendi impotence and the possible recurrence of a fistula [7,8]. The angiographic examination made it possible to obtain a more detailed evaluation of the lesion’s anatomical configuration and to choose the best therapeutic strategy to employ [9]. The adopted two phase strategy permitted complete treatment of the malformation, which was large-sized and was served by 4 main blood confluences. The embolization caused a progressive reduction in the agglomerate, and this allowed surgical excision with a minimum risk of bleeding, as well as the surgical removal of the necrotic scrotal skin area. Moreover, it was possible to preserve the integrity of the didymus located near the lesion and avoid the development of Fournier’s gangrene. The agglomerate decreased in size until near complete restoration (“restitutio ad integrum”) was achieved. A later reconstructive plastic surgery procedure was performed to treat the entire area affected by the lesion [10] (Fig. 3C).

## 4 Conclusions

Scrotal malformations are quite rare, and they can involve problems related to the therapeutic strategy chosen. The multidisciplinary approach used in this case involved a less invasive treatment protocol and subsequently less risky surgery. This strategy allowed the complete exclusion of the malformation and the retention of erectile capacity but did not improve organ function, a goal that remained unmet. To obtain a conclusive evaluation, an additional period of time is required to monitor the regression of the tumefaction in the long term as well as the aesthetic results and eventual benefits regarding fertility.

## Funding

No funding was declared and the acknowledgement are reported at the end of the text. Authors declare that the sponsor have no role in the study, in the collection or analysis and interpretation of data; in the writing of the manuscript; and in the decision to submit the manuscript for publication.

## Ethical approval

The patient give the consensus to the procedure. Ethical approval is exempt for case reports by our institution.

## Consent

Written informed consent was obtained from the patient for publication of this case report and accompanying images. A copy of the written consent is available for review by the Editor-in-Chief of this journal on request.

## Author contribution

Study concept or desig: AAM, GC, RG and AI.

Data collection: AAM, GC, RG and AI.

Data analysis or interpretation: AAM, GC, RG and AI.

Writing the paper: AAM, GC, RG and AI.

## Registration of research studies

This publication is free from Registration of Research Studies, because is not used a new technology.

## Guarantor

Prof. Arnaldo Ippoliti, Prof. Andrea Ascoli Marchetti.

## Provenance and peer review

Not commissioned, externally peer-reviewed.

## Declaration of Competing Interest

The authors declare to have no conflicts of interest or any financial and personal relationship with other people or organisations that could inappropriately influence (bias) our work.

## References

[1] Agha R.A., Borrelli M.R., Farwana R., Koshy K., Fowler A., Orgill D.P., For the SCARE Group (2018). The SCARE 2018 Statement: Updating Consensus Surgical CAse REport (SCARE) Guidelines. Int. J. Surg..

[2] Jacobowitz G.R., Rosen R.J., Rockman C.B., Nalbandian M., Hofstee D.J., Fioole B., Adelman M.A., Lamparello P.J., Gagne P., Riles T.S. (2001). Transcatheter embolization of complex pelvic vascular malformations: results and long-term follow-up. J. Vasc. Surg..

[3] Calligaro K.D., Sedlacek T.V., Savarese R.P., Carneval P., DeLaurentis D.A. (1992). Congenital pelvic arteriovenous malformations: long-term follow-up in two cases and a review of the literature. J. Vasc. Surg..

[4] Rastogi R. (2008). Diffuse cavernous Hemangioma 1 of the penis, scrotum, perineum and rectum - a rare tumor. Saudi J. Kidney Dis. Transpl..

[5] Monoski M.A., Gonzalez R.R., Thomas A.J., Goldestain M. (2006). Arteriovenous malformation of scrotum causing virtual azoospermia. Urology.

[6] Minei S., Minamida S., Dobashi M., Ishii J., Minei S., Irie A. (2008). Varicocele complicating spontaneous arteriovenous fistula. Int. J. Urol..

[7] Agrawal V., Dangle P., Minhas S., Ralph D., Christopher N. (2006). Recurrent arteriovenous malformation of the scrotum secondary to pelvic trauma. Urol. Int..

[8] So W.L., Chaganti J., Waugh R., Ferguson R.J. (2015). Management of scrotal arteriovenous malformation with transcatheter embolisation coils and percutaneous sclerotherapy under angiographic guidance. J. Med. Imaging Radiat. Oncol..

[9] Romagnoli A., Bertolotto F., Carmignani G. (2004). An usual varicocele due to spontaneous arteriovenous fistula. Urology.

[10] Cervelli V., Brinci L., Palla L., Spallone D., Izzo V., Curcio C.B., Lucarini L., De Angelis B. (2012). Skin necrosis of scrotum due to endovascular embolisation: a case report. Int. Wound J..

